# Detection of Pesticide Residue Level in Grape Using Hyperspectral Imaging with Machine Learning

**DOI:** 10.3390/foods11111609

**Published:** 2022-05-30

**Authors:** Weixin Ye, Tianying Yan, Chu Zhang, Long Duan, Wei Chen, Hao Song, Yifan Zhang, Wei Xu, Pan Gao

**Affiliations:** 1 College of Information Science and Technology, Shihezi University, Shihezi 832003, China; yeweixin@stu.shzu.edu.cn (W.Y.); yantianying@163.com (T.Y.); duanlong@stu.shzu.edu.cn (L.D.); chenwei@stu.shzu.edu.cn (W.C.); songhao@stu.shzu.edu.cn (H.S.); 2 School of Information Engineering, Huzhou University, Huzhou 313000, China; chuzh@zjhu.edu.cn; 3 College of Agriculture, Shihezi University, Shihezi 832061, China; zyf1005225469@163.com; 4 Xinjiang Production and Construction Corps Key Laboratory of Special Fruits and Vegetables Cultivation Physiology and Germplasm Resources Utilization, Shihezi 832003, China

**Keywords:** hyperspectral imaging, pesticide residue, table grape, deep learning, non-destructive detection

## Abstract

Rapid and accurate detection of pesticide residue levels can help to prevent the harm of pesticide residue. This study used visible/near-infrared (Vis-NIR) (376–1044 nm) and near-infrared (NIR) (915–1699 nm) hyperspectral imaging systems (HISs) to detect the level of pesticide residues. Three different varieties of grapes were sprayed with four levels of pesticides. Logistic regression (LR), support vector machine (SVM), random forest (RF), convolutional neural network (CNN), and residual neural network (ResNet) models were used to build classification models for pesticide residue levels. The saliency maps of CNN and ResNet were conducted to visualize the contribution of wavelengths. Overall, the results of NIR spectra performed better than those of Vis-NIR spectra. For Vis-NIR spectra, the best model was ResNet, with the accuracy of over 93%. For NIR spectra, LR was the best, with the accuracy of over 97%, but SVM, CNN, and ResNet also showed closed and fine results. The saliency map of CNN and ResNet presented similar and closed ranges of crucial wavelengths. Overall results indicated deep learning performed better than conventional machine learning. The study showed that the use of hyperspectral imaging technology combined with machine learning can effectively detect the level of pesticide residues in grapes.

## 1. Introduction

Grapes are one of the most popular fruits due to its unique taste, multiple vitamins, and nutrients. Grapes can be eaten fresh and processed into various products, for instance, juice and wine. Thus, there exists excellent commercial potential for the grape industry. During the grape growing season, fungicides, insecticides, and herbicides are often applied to cure the stresses of the diseases and pests [1,2]. The pesticide residue in grapes has increasingly aroused the attention of consumers. Certain intake of pesticide residue content may harm consumers’ health [3,4].

Various methods have been developed for the detection of pesticide residue in fruits and vegetables [5]. Generally speaking, they can be divided into conventional and rapid detection methods. Traditional detection methods for detecting pesticide residues include gas chromatography (GC) and capillary electrophoresis (CE) [6], gas chromatography-mass spectrometry (GC-MS) [7], high-performance liquid chromatography (HPLC) [8], supercritical fluid chromatography (SFC) [9], and so on. Rapid detection methods include the fast detection card method and enzyme inhibition rate method. These methods have high accuracy for the detection of pesticide residue. However, they are costly. Moreover, they require complex pre-processing and highly skilled operators.

Hyperspectral imaging (HSI) is a technology that combines spectroscopy and conventional imaging to attain the spectral and spatial information from the research object [10]. HSI has been used effectively in the non-destructive quality detection of grapes, such as total soluble solids [11,12,13], total phenolic compounds [12], polyphenol contents [14], amino acids [11], and PH [11,12], etc. Moreover, there have been quantitative analyses, such as discriminating geographical origin [15], the year of harvest [15,16], and the maturation stage [17], etc.

Detection of pesticide residue in agricultural products combined with HSI technology has also been used widely, due to its advantage of rapid, non-destructive, and accurate quality detection. Sun et al. used HSI technology (431–962 nm) to quantitatively identify the pesticide mixtures on lettuce leaves [18]. Jia et al. detected apple surface pesticide residue based on HSI technology (865–1712 nm) [19]. Mohite et al. used hyperspectral sensing (350–1052 nm) to detect pesticide (Cyantraniliprole) residue on grapes with no, single, and double doses [20]. Ren used HSI technology (900–170 nm) to distinguish various concentrations of pesticide residues of dimethoate on the surface of spinach leaves [21]. Sun et al. identified pesticide residues in lettuce combining chemical molecular structure and NIR hyperspectral (870–1780 nm) [22]. Jiang et al. used NIR HIS (390–1050 nm) to predict the distribution of pesticide residues on mulberry leaves and visualize the results [23]. Studies have shown that HSI technology has been widely used in the non-destructive detection of pesticide residue in agricultural products. However, research on the pesticide residue in grapes is still rare, and a single spectral region was studied for most. Therefore, it is feasible and proposed to use hyperspectral imaging technology to detect different levels of pesticide residues in grapes here.

It is a great challenge to research massive and redundant data obtained by hyperspectral imaging systems (HIS) effectively, which prevents its application. Machine learning is exceptionally crucial for predicting features and analyzing spectral information. Recently, deep learning, as a new method of machine learning, has gained remarkable results for detecting and classifying the spectral and spatio-spectral signatures in HIS. Deep learning learns features deeply and automatically, and processes large volumes of data effectively [24,25,26]. Thus, it can construct a network containing many neurons efficiently and quickly, and it is applied widely in spectroscopy [27,28,29,30]. Yan et al. used HIS with deep learning to detect geographical origin of Radix Glycyrrhizae [31]. Jiang et al. used HIS with AlexNet-CNN deep learning network to detect postharvest pesticide residues [32]. Dreier et al. used CNN and ResNet with HSI to identify the bulk grain [33]. Gomes et al. used deep learning CNN to predict sugar and pH levels in grapes [34]. Deep learning has decent performance, but the process is obscure and difficult to understand. The contribution of wavelength is visualized to observe crucial wavelengths, which can explain the deep learning process well and analyze data effectively.

The purpose of the study was to use hyperspectral imaging technology combined with machine learning to identify the different pesticide residue levels in grapes. The specific goals were: (1) to explore the spectral differences among different pesticide residue levels of different varieties of grape; (2) to compare the performances of hyperspectral imaging at two different spectral regions for pesticide residue level identification; (3) to compare the performances of conventional machine learning methods (LR, SVM, and RF) and deep learning (CNN and ResNet); (4) and to explore the spectral features of different models which contribute more to the identification.

## 2. Materials and Methods

### 2.1. Samples Preparation

**Figure 1:**
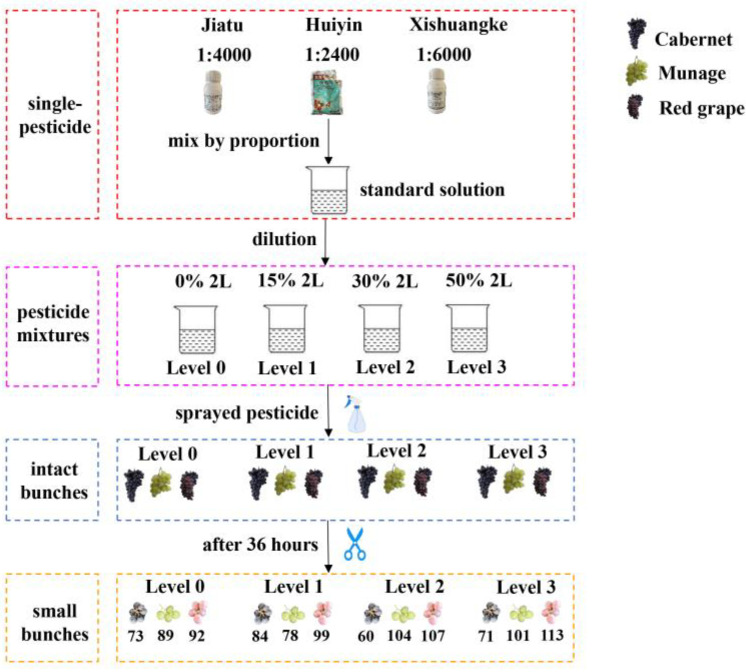
The flow chart of spraying pesticides and obtaining clusters of the grape.

The research was carried out in the laboratory and simulated the process of spraying pesticides. Three grape varieties were used in this study, including Munage, Cabernet Sauvignon (Cabernet), and Red grape. The fresh grapes of Munage were purchased from the Jinma Market near Shihezi University, and Cabernet and Red grape were collected from the experimental vineyard located in the School of Agriculture, Shihezi University, Xinjiang Uygur Autonomous Region (Xinjiang), China (73°40′–96°18′ E, 34°25′–48°10′ N). Each grape variety was randomly divided into four groups, corresponding to four different concentrations of pesticide residues (corresponding to four levels mentioned later). To increase the number of samples and comply with sampling inspection in the actual production, the bunch of the grape was cut smaller, considering the cluster of 3–6 berries as a sample, as shown in Figure 1. After cutting off grape bunches, 288 clusters of Cabernet, 411 clusters of Red grape, and 372 clusters of Munage were collected. In total, 1071 small clusters of grapes were used as input samples. The sample data were randomly divided into training, validation, and test sets with a ratio of 3:1:1. The specific sample size of clusters of the grape is shown in Table 1.

In this study, Jiatu (25% trifloxystrobin, 50% tebuconazole), Xishuangke (56% cymoxanil, 14% cyazofamid), and Huiyin (80% procymidone) were prepared, and the details are shown in Table 2. According to relevant information and instructions, these pesticide mixtures do not react chemically but only enhance the effect. Pesticide mixtures were sprayed on the grapes to simulate the pesticide residue. Different pesticides were applied to evaluate their effects on the growth of the grape. One reason for choosing these three pesticides was wide use during the ripening period of the grapes, and the other was the recommendations and suggestions of the planter. Roughly speaking, Jiatu, Xishuangke, and Huiyin are common fungicides, and they have a certain inhibitory effect on the growth of fungi.

There were two steps to making pesticides mixtures:

(1) Make standard pesticide mixtures. According to the instructions of each pesticide, three single-pesticide solutions (Jiatu, Huiying, and Xishaungke) were prepared with the proportion of 1:4000, 1:6000, and 1:24,000, respectively. Then, the three single-pesticide solutions were mixed together to make a 2 L pesticide mixture, as 100% standard pesticide mixtures.

(2) Make three pesticides mixtures. A beaker was used to dilute the 100% standard solution into three different pesticide mixtures. Concentrations of three pesticide mixtures were 10%, 15%, and 50% (respectively corresponding to Level 1, Level 2, and Level 3). Level 0 represented distilled water as a control group for comparing with others.

The corresponding concentration of the final configuration of each pesticide is shown in Table 3.

With a spraying bottle, four groups of grapes were sprayed with Level 0, 1, 2, and 3 mixed pesticides, respectively. Then, the sprayed grapes were placed in a low-temperature and ventilated area for air drying for about 36 h [18,23,35,36,37]. When there was no more water on the grape surface, each intact bunch of grapes was cut, as shown in Figure 1.

### 2.2. Hyperspectral Image Acquisition and Correction

In this study, Vis-NIR and NIR HISs (SOC 710VP and SOC 710SWIR) were used in obtaining hyperspectral images. The SOC 710VP covers the spectral range of 376–1044 nm (128 bands), captures the image size of each waveband with 520 pixels × 696 pixels, and has an exposure time of 24 ms and a spectral resolution of 5 nm. The SOC 710SWIR covers the spectral range of 915–1699 nm (288 bands), captures the image size of each waveband with 512 pixels × 640 pixels, and has an exposure time of 34 ms and a spectral resolution of 2.7 nm. The distance from the sample to the imaging device was adjusted to 93.5 cm. Other information about the two HISs can be shown in Yan [31]. In the study, grapes of Level 0, Level 1, Level 2, and Level 3 were captured sequentially by the HIS. Each sample was fully photographed by shooting the front and back (randomly, one side was the front, and the other side was the back).

The raw hyperspectral images were corrected into the reflectance images by using a grayscale reference image. The correction was conducted by the following Equation (1):

(1) $$I_{r}=\frac{I_{raw}-I_{dark}}{I_{white}-I_{dark}}$$

$I_{r}$ is the reflectance image, $I_{raw}$ is the raw image, $I_{white}$ is the entirely white reference image, and $I_{dark}$ is the entirely black reference image. The grayscale reference image was composed of 50% $I_{dark}$ and 50% $I_{white}$.

### 2.3. Spectral Data Preprocessing and Extraction

**Figure 2:**
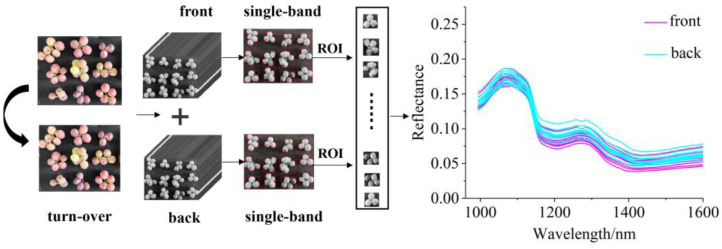
The flow chart of hyperspectral image data acquisition and data contact.

The segmentation between the grape and the background was necessary to obtain accurate spectral information. In this study, ENV 5.2 (ITT Visual Information Solutions, Boulder, CO, USA) was used to crop a hyperspectral image to various hyperspectral sub-images containing a sample of 3–6 single berries. The sample in each hyperspectral sub-image was defined as a region of interest (ROI), which is a mask formed by threshold segmentation of the 804 nm Vis-NIR hyperspectral sub-image and the 1092 nm NIR hyperspectral sub-image. Further, spectra information in the ROI of the hyperspectral sub-image was extracted by Matlab R 2018b (The Math Work, Natick, MA, USA). The average spectrum of ROI was calculated as the spectral value of the sample, as shown in Figure 2. The spectral value at the beginning and the end were removed to eliminate obvious noise. The reserved wavelength range of Vis-NIR spectra was 476–890 nm (80 bands), and that of NIR spectra was 970–1594 nm (230 bands). For Vis-NIR and NIR spectral value, Savitzky–Golay (SG) [38] smoothing filter (the polynomial order was 0, the kernel size was 3) was used to improve the smoothness of the spectra and reduce noise interference. Then, the Standard Normal Variate transform (SNV) [39] was applied to avoid the impact of surface scattering, solid particle size, and the optical path change of diffuse reflection spectra.

### 2.4. Data Analysis Method

#### 2.4.1. Principal Component Analysis (PCA)

Principal component analysis (PCA) is a commonly used statistical method. A group of variables related to each other can be transformed into uncorrelated and independent ones through orthogonal transformation [40,41]. The primary purpose is to reduce the number of variables, namely dimensionality reduction. It is a linear dimensionality reduction method. The transformed variable is called the principal component (PC), and the top PCs explain most of the information of the hyperspectral image. The PCA score scatter plots for qualitative analysis of grape pesticide residues could be formed.

#### 2.4.2. Support Vector Machine (SVM)

Support vector machine (SVM) is a supervised pattern recognition approach. SVM is a traditional classification method, and it is widely applied in classification conditions [42,43]. Moreover, SVM has excellent generalization ability, so it is widely used in spectroscopy. The kernel function is highly vital to the SVM model. In this paper, the tuning range of the kernel function was “poly, rbf, sigmoid”. The kernel parameter g and penalty coefficient C were used to get optimal performance. A grid-search procedure was used to optimize *g* and *C*. The searching range of *g* and *C* were 10^−5^ to 50 and 10^−5^ to 50, respectively.

#### 2.4.3. Logistic Regression (LR)

Logistic regression (LR) is a generalized linear regression analysis model, and it is often used in data mining, automatic disease diagnosis [44], economic forecasting [45], and other fields [46]. Linear regression is a machine learning method used to solve binary classification (0 or 1) problems, which are used to estimate the possibility of something. Adding the sigmoid active function to linear regression, LR can then be used for multiple classifications and introduced non-linear elements [47]. In this study, the optimization range of the solver was in ‘‘newton-cg’’, ‘‘lbfg’’, ‘’liblinea’’, ‘’sag’’, and that of C was between 10^−5^ and 10^5^. The penalty was set to L2.

#### 2.4.4. Random Forest (RF)

RF is ensemble learning, which consists of the decision tree (DT) [48]. RF shows two important traits: random sampling of training data points when building trees, and random subsets of features considered when splitting nodes [49,50]. The last result of the decision is determined by the voting method, so it has strong robustness. Random forest can process high-dimensional data without feature selection. In our study, *n_estimators* were between 100 and 1000, and *max_depth* was between 4 and 8.

#### 2.4.5. Convolutional Neural Network (CNN)

**Figure 3:**
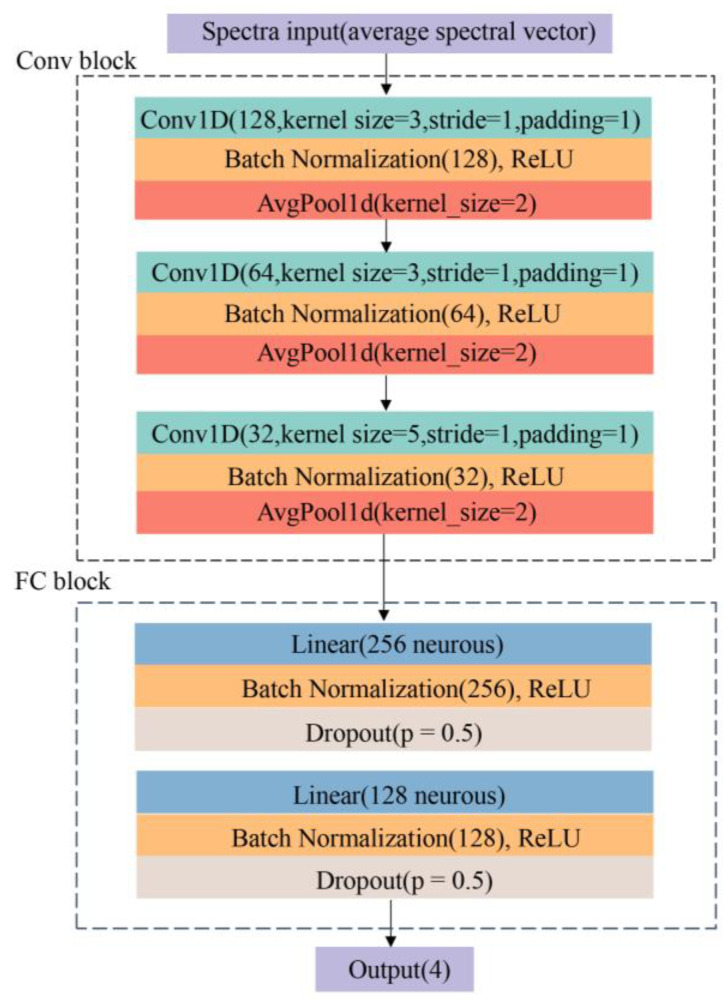
The proposed convolutional neural network (CNN) structure for the identification of pesticide residues in grapes.

A convolutional neural network (CNN) is a forward neural network. It usually consists of the following six layers: input layer, convolution layer, activation layer, pooling layer, fully connected layer, and output layer [31]. CNN has an excellent performance in classification. One advantage of CNN is local perception. CNN only perceives the local elements of the data and then merges local information in the higher-level network to obtain all the characterization information of the data. The second is weight sharing. By weight sharing, the number of weights of the network can be decreased, and the complexity of the network can be reduced [29]. A simple CNN architecture was designed for our study. The structure of the CNN is shown in Figure 3.

In Figure 3, two main blocks were involved in the structure. The first block was the convolutional block (Conv Block), which consisted of three convolutional layers. Each convolutional layer was followed by a batch normalization layer (BN) and rectified linear unit (ReLU). In the end, an average pooling layer was added to alleviate the excessive sensitivity from the convolutional layer. In this process, one-dimensional (1D) spectral data were involved, and Conv1D was used, as shown in Figure 3. The second was a fully connected block (FC Block). The features extracted by the convolutional layer were learned through the fully connected layer. A linear layer was added, and BN and ReLU followed. The dropout was applied to alleviate overfitting. For the output layer, the network outputs the final result according to the probability of the four classification results. The input channels of the first three convolutional layers were 128, 64, and 32; the kernel sizes were 3, 3, and 5; the stride was 1, and the padding was 1. For the average pooling layer, kernel size was 2. The FC block included two fully connected layers, which consisted of 256 and 128 neurons, respectively. The dropout ratio was set as 0.5. Another linear layer was set for output at the end of the network. During the training process of CNN, the Adaptive Moment estimation (Adam) algorithm was used to optimize softmax cross-entropy. Weights were initialized using the Xavier algorithm.

#### 2.4.6. Residual Neural Network (ResNet)

**Figure 4:**
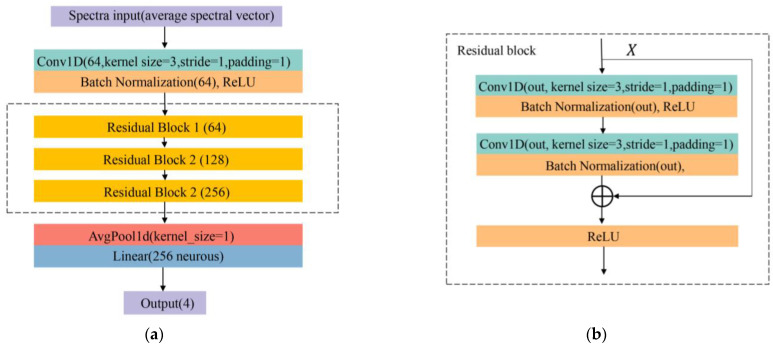
The proposed residual neural network (ResNet) (**a**) and residual block (**b**) structures for the identification of pesticide residues in grapes.

With the deepening of the neural network, there would be problems of overfitting, gradient explosion, and network degradation, and ResNet could effectively handle those [51]. In this study, based on the ResNet18, the ResNet was applied to identify pesticide residual levels. Figure 4a shows the structure of ResNet. The ResNet consisted of one convolutional layer and two residual blocks, the last was average pooling. The output channel of the convolutional layer was 64, kernel size was 1 × 3, and stride and padding were 1. Then a batch normalization layer (BN) and rectified linear unit (ReLU) were added. The channels of 3 residual blocks were 64, 128, and 256, kernel size was 1 × 3, and stride and padding were 1. The average pooling was followed to extract features smoothly, the last was the linear layer.

### 2.5. Saliency Map

Saliency map is a visualization technique in order to gain better insights into the decision-making of a neural network. When a sample was predicted correctly, it would be added to compute the feature importance [52]. Scale information contributions within the network could be computed [53]. Once the sample label was correctly predicted, the corresponding weights of the elements would be obtained, which represents the contribution rate (importance) of the elements. A saliency map can visualize the contribution rate of each element to intuitively see which elements play important roles in the process of CNN-based sample identification. For hyperspectral data, a saliency map could effectively visualize the importance of the wavebands.

Given the hyperspectral data *X*_0_ with the set of the test, which was built by the classification model CNN-based, the class score function *S*_C_ (*X*_0_) was obtained for all the wavebands [53]. When the label of this sample was correctly classified, the weight *w* could be calculated by the followed Equation (2).

(2) $$\;\;w=\mathrm{abs}\left(\frac{\partial S}{\partial X}|X_{0}\right)$$

where *w* means the absolute value of the derivative of the score value *S* concerning the spectral data *X*_0_.

In this study, test set data were used to compute the importance of all the wavelengths, when the sample label was predicted correctly.

### 2.6. Software and Model Evaluation

In this study, the areas of the samples were defined in ENVI 5.2 (ITT Visual Information Solutions, Boulder, CO, USA). The spectral data were extracted in Matlab R 2018b (The Math Work, Natick, MA, USA). The Python scripting language (version 3.8,64 bit) was applied for the numerical calculations. SVM, LR, and PLS-DA were conducted by using the machine learning library scikit learn (version 0.23.2). The 1D CNN model was built on the deep learning Pytorch framework (version 1.5.1). All data analysis procedures were implemented on a computer with a memory of 10 GB, a SSD of 238.35 GB, and a CPU of i5-7200 U.

The accuracy is used to illustrate the discrimination ability of classifier systems. The definition was the following:

(3) $$Accuracy=\frac{TP\;}{All\;}$$

*TP* (true positive) means the number of the predicted result consistent with the actual label. *All* means the number of all samples. *Accuracy* is the index to evaluate the model.

## 3. Results

### 3.1. Spectral Profiles

**Figure 5:**
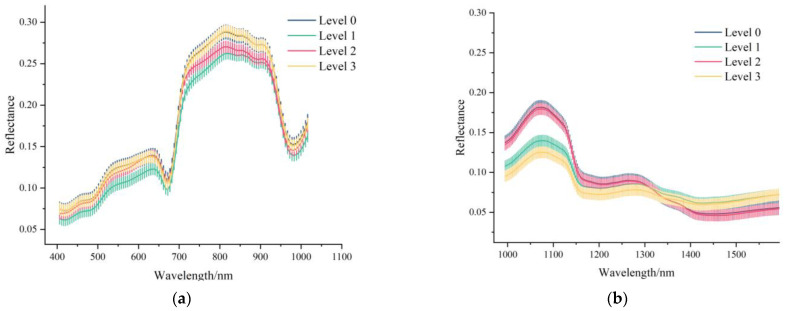
(**a**) Vis-NIR average (405–1016 nm) spectra with standard deviation each wavelength of different levels of pesticide residues in grape, using Vis-NIR spectrometer. (**b**) NIR average spectra (994–1641 nm) with standard deviation each wavelength of different levels of pesticide residues in grapes, using NIR spectrometer.

The spectra in the range of 376–1073 and 915–1699 nm were extracted from the Vis-NIR and NIR HISs. The beginning and end of the spectra showed obvious noises. The spectral data were preprocessed by SG. The average spectra of four pesticide mixture levels and corresponding standard deviation are shown in Figure 5.

According to Figure 5, it is clear that the trend of the four average spectral curves is mostly similar. Peaks and valleys exist in the certain same positions and have no overlap (around 825, 550 and 1725 nm), which might have the potential to identify the different levels of pesticide residue in grapes due to variation of spectral reflectance in Vis-NIR and NIR regions. However, different pesticide levels and spectral ranges showed some discrepancies. In Figure 5a, the error bar overlaps at almost the entire band, and the curves of average spectra intersect at about 690 nm and 950 nm. In Figure 5b, the error bar overlaps in the spectra between 1160 nm and 1490 nm, and curves of average spectra intersect at 1310 nm. Therefore, it is impossible to directly distinguish different levels of pesticide residues in grapes clearly. It is necessary and crucial to do further research.

### 3.2. Principal Component Analysis (PCA)

To preliminarily explore significant differences between four levels of pesticide residues in grapes, spectral data were analyzed based on PCA. The two-dimensional PCA score plots were shown in Figure S1, with the sample’s distribution of each PC. The corresponding confidence ellipse was added, with a confidence level of 0.95.

For Vis-NIR spectra, the contributions of the first three PCs of Cabernet were 48.5%, 27.5%, and 10.0%; those of Red grape were 49.4%, 26.8%, and 12.7%; those of Munage were 71.0%, 13.3%, and 4.6%. Their cumulative contributions of them were, respectively, 86.0%, 88.9%, and 88.9%, which explained most of the sample. However, the PCA score plots were clustered badly and there was serious overlap. For Cabernet, in Figure S1a–c, distributions of PC1 versus PC2, PC1 versus PC3, PC2 versus PC3 are chaotic and huddled, which means the four levels of pesticide residue are indistinguishable from each other. This phenomenon is consistent with trends of the spectral profile in Figure 5a. In addition, there is a certain similarity in Figure S1d–i.

For NIR spectra, the contributions of the first three PCs of Cabernet were 56.3%, 22.3%, 17.5%; those of Red grape were 57.4%, 31.3%, 6.6%; and those of Munage were 70.8%, 20.0%, 4.9%. The cumulative contributions of the first three PCs were 96.1%, 95.3%, and 95.7%, respectively, which also explained most of the variance information. Regarding the sample distribution, the overall clustering effect was slightly better than that of the Vis-NIR. For Cabernet, in Figure S1j–l, two major aggregating regions were shown (Level 0 and Level 2, Level 1 and Level 3), which is consistent with the phenomenon in Figure 5b. Therefore, the result comparatively illustrates the feasibility of the identification of four levels of pesticide residues in the range of NIR spectra.

In general, PCA visualizes sample distribution and provides the feasibility of classification, but it is not easy to directly distinguish the four levels of pesticide residues. Therefore, it is necessary to find other multivariate analysis methods for further research.

### 3.3. Classification Models

Three machine learning algorithms (SVM, LR, and RF) and two deep learning (CNN and ResNet) algorithms were conducted to analyze spectral data in this stage. The results are shown in Table 4 below.

**Vis-NIR spectra**. All the models had good performances and had an average accuracy of over 90% for training, validation, and prediction sets. For Cabernet, the best models, the CNN and ResNet models, showed closed results, with the accuracy of over 99%, 94%, and 93% for train, validation, and test sets. SVM and LR models showed closed results, with the accuracy of over 91%, 89%, and 100% for training, validation, and test sets. For Red grape, all the models showed an accuracy of over 90% for training, validation, and test sets. RF showed overfitting, with the accuracy of over 100%, 77%, and 79%. For Red grape, the best model was ResNet, with the accuracy of over 100%, 100%, and 98% for training, validation, and test sets. CNN, SVM, and LR were slightly lower, with the accuracy of 97%, 96%, and 92% for training, validation, and test sets. RF still showed overfitting, with the accuracy of 99%, 72%, and 73% for training, validation, and test sets. For Munage, the best model was ResNet, with the accuracy of 100%, 97%, and 94% for training, validation, and test sets. CNN was slightly lower, with the accuracy of 100%, 98%, and 94% for training, validation and test sets. SVM performed with an accuracy of 100%, 88%, and 93.2% for training, validation, and test sets. RF was inferior to others, with the accuracy of 100%, 66%, and 75% for training, validation, and test sets. Overall, there was no significance with a different variety. ResNet performed better than other models, RF showed the overfitting, and SVM, LR, and CNN presented the fine result.

**NIR spectra**. Generally, all models had a slightly better result than Vis-NIR spectra, SVM, LR, CNN, and ResNet showed the average accuracy of over 90% for the validation set. For Cabernet, the CNN, LR, and SVM models presented the best and similar results, with an accuracy of close to 96% of the validation set. The following was ResNet, with the accuracy of 100%, 93%, and 86% for training, validation, and test sets. RF showed overfitting, with the accuracy of 100%, 74%, and 81% for training, validation, and test sets. For Red grape, SVM, LR, CNN, and ResNet presented closed and fine results, with the accuracy of over 100%, 100%, and 96% for training, validation, and test sets. RF showed lower results, with the accuracy of 98%, 86%, and 87.8% for training, validation, and test sets. For Munage, all models presented decent results, with the accuracy of over 93%. Overall, all the models showed fine results, and the results performed better than those of Vis-NIR. RF still showed the overfitting for Red grape and Munage. Varieties were not significant in the three grapes.

**Methods**. Considering different methods, there was a slight difference. For Vis-NIR spectra, overall, ResNet was the best model, with the accuracy of over 100%, 94%, and 93% for training, validation, and test sets. The following was CNN, with the accuracy of over 97%, 97%, and 92% for training, validation, and test sets. SVM and LR model were closed, with the accuracy of over 91% for the validation set. RF showed overfitting. For NIR spectra, SVM, LR, CNN, and ResNet showed closed and fine results, with an average accuracy of over 90%, but RF also showed overfitting for Cabernet. Overall, the deep learning methods (CNN, ResNet) performed better and had more stable results than those of machine learning (SVM, LR, RF).

The overall classification results showed NIR spectra performed better than Vis-NIR spectra. HIS in the NIR region was attributed to the overtone and overtone combination of molecular bonds (e.g., N-H, C-H, and O-H), and HIS in the Vis-NIR region was related to object color (e.g., chlorophyll). The results showed that spectral information on pesticide residues was related to the overtone of molecular, and more valuable information would be extracted via NIR spectra than Vis-NIR spectra regarding pesticide residue in grape. Therefore, it was more suitable to detect pesticide residues using NIR spectra. For Vis-NIR, CNN and ResNet performed best. For NIR, all results performed equally well, with the accuracy of over 95%. Overall, it shows that the deep learning method is superior to the traditional method. However, RF showed overfitting, and the reason might be the small size of the sample. The results of each grape variety showed a consistent trend. Thus, the classification accuracy did not correlate with the grape variety.

### 3.4. Visualization for Discovering the Wavelength Importance

**Figure 6:**
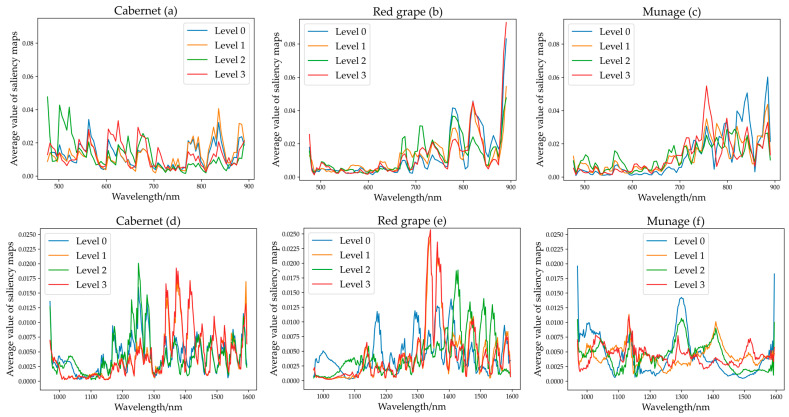
(**a**–**c**) Mean average value of saliency map of CNN for Cabernet, Red grape, and Munage for Vis-NIR spectra. (**d**–**f**) Mean of CNN for Cabernet, Red grape, and Munage for NIR spectra.

**Figure 7:**
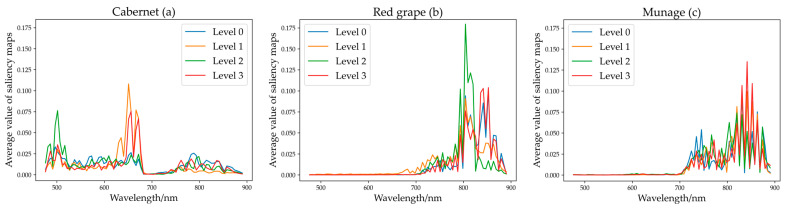

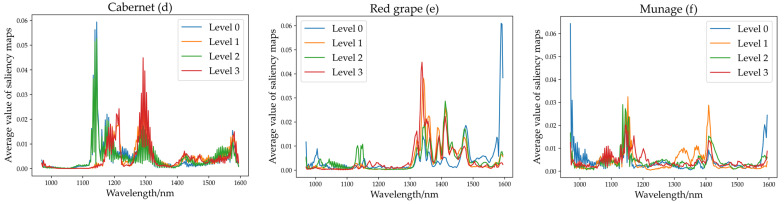
(**a**–**c**) mean average value of saliency map of ResNet for Cabernet, Red grape, and Munage for Vis-NIR spectra. (**d**–**f**) mean that of ResNet for Cabernet, Red grape, and Munage for NIR spectra.

Overall, deep learning (CNN and ResNet) offered finer results than machine learning, but their process of operation is hard to interpret. Therefore, CNN and ResNet were selected to visualize the wavelength importance, and saliency map was applied to analyze the model to find the critical wavelengths. The data were processed with normalization. The larger the value of the saliency map, the more critical the wavelength. The results are shown in Figure 6 for CNN and Figure 7 for ResNet.

**Saliency map of CNN.** For Vis-NIR spectra of Cabernet, approximately 500–530 nm, 550–580 nm, 600–730 nm, and 760–900 nm showed the largest contribution, and the difference between all bands was not very significant. For Vis-NIR spectra of Red grape and Munage, there were similar trends, approximately 660–900 nm contributed the most. For NIR spectra of Cabernet and Red grape, there was a consistent trend, approximately 1150–1300 nm and 1320–1600 nm contributed the most, the others showed low contribution. For NIR spectra of Red grape, regions of large contribution rate were 1290–1600 nm and 1120–1195 nm. For the NIR spectra of Munage, regions of main contribution were 960–1080 nm, 1110–1150 nm, 1280–1320 nm, 1390–1460 nm, and 1500–1550 nm.

**Saliency map of ResNet.** For the Vis-NIR spectra of Cabernet, the wavelengths at approximately 470–530 nm and 650–690 nm contributed the most, followed by the wavelengths at approximately 530–650 nm and 750–880 nm. For the Vis-NIR spectra of Red grape and Munage, the results presented the similarity; the wavelength at approximately 710–900 nm contributed the most. For the NIR spectra of Cabernet, the wavelengths at approximately 1120–1210 nm and 1260–1310 nm contributed the most, followed by the wavelengths at approximately 1210–1310 nm and 1420–1600 nm. For NIR spectra of Red grape, the wavelengths at approximately 1300–1500 nm and 1590 nm contributed the most, the others were low. For the NIR spectra of Munage, the wavelengths at approximately 970–980 nm, 1130–1180 nm, 1400–1420 nm, and 1580–1600 nm contributed the most, followed by the wavelengths at approximately 980–1110 nm and 1300–1440 nm.

For Vis-NIR spectra, generally, wavelengths of 380–780 nm were mainly relevant to the color variations of grape, e.g., chlorophyll [12,54]. For the rest of the NIR regions between 780 and 900 nm, those wavelengths are attributed to the third overtone stretch of O-H related to water in grapes [55]. The range of 900–980 nm was contributed to by the third overtone of C-H relevant to sugar [55]. For NIR spectra, wavelengths between 1050 nm and 1200 nm are mainly made up of the second overtone of C−H, and those between 1300 nm and 1500 nm are mainly related to the frequency of C-H [56]. The range of 1210–1450 nm is attributed to the 2nd overtone of C-H and the 1st overtone of O-H [54]. The wavelength between 975 nm and 1015 nm is mainly attributed to N-H stretch second overtone [57], and 1526 nm (N-H stretch first overtone) [58], which can reflect pesticide residue differences among different levels. Since Jiatu, Huiyin, and Xishuangke contain a large amount of C-H, O-H, and N-H as observed by their chemical molecular formula, these selected bands have a great correlation with pesticides. Overall, the saliency map of CNN and ResNet showed a similar and consistent trend, which confirmed the feasibility of visualizing the contribution of wavelengths by this method.

## 4. Discussion

Visible/near-infrared spectroscopy or hyperspectral imaging is a fast and non-destructive method to detect pesticide residues. Some studies have applied HSI to detect pesticide contaminants in foods [9,19,20,21,22], but the research object in those experiments was single and lacked mutual comparison between the objects. In our study, we chose three grapes to identify the difference between the varieties. Moreover, those studies used one [23] or two [18] pesticides as solvents for the research, and few studies mixed pesticides. Generally speaking, mixed pesticides can more effectively control plant diseases and insect pests without affecting the chemical properties and structure of the active ingredients. In this study, we used three pesticides together (Jiatu, Huiyin, and Xishuangke) to make the pesticide mixture and set four levels to compare the correlation among them, which was more consistent with the actual production with the use of pesticide. In addition, other studies mainly focused on assessing the pesticide residue within a single spectral range, and there is rarely a combination of Vis-NIR and NIR used on pesticide residues. In particular, to the best of our knowledge, no attempts have been made to analyze the different spectral ranges of mixed pesticides in grapes. Two spectral ranges were chosen to form a contrast and study the difference between the spectra, which increases the range of the spectrum and makes the research more comprehensive.

Due to the redundancy and high volumes of hyperspectral data, machine learning and deep learning were used to process the data and extract features. Previous studies have used SVM [22,59], DT [59], KNN [59], or RF [18] to detect pesticide residue, which showed fine results. SVM [60,61,62], LR [63], CNN [31,56,64], RF [60,62], and ResNet [56] have been applied widely in quality detection of hyperspectral imaging. In this study, classic machine learning and deep learning methods, CNN, ResNet, LR, SVM, and RF, were used to achieve a multivariate analysis of the detection of pesticide residue levels in grapes. More importantly, the saliency maps of CNN and ResNet were conducted to visualize the contribution rate of the wavelength, which brought us a clear understanding of the crucial wavelength information.

The two spectral ranges of the Vis-NIR (376–1044 nm) and NIR (915–1699 nm) showed great potential and decent results for detecting pesticide residue at different levels. The results (Table 4) of the NIR spectra were slightly better than those of the Vis-NIR spectra, with average accuracies of 95% and 90%, respectively. However, in this study, the main challenge was to make pesticide mixtures well-distributed in grapes. The uneven spraying of pesticides has a profound impact on the reflectance of hyperspectral images. The brightness unevenness of the hyperspectral image caused by the change in the surface curvature of the sphere also needs to be carefully corrected. The time-varying nature of spectrum acquisition deserves attention, such as drying time and acquisition sequence. The study promotes the non-destructive detection of pesticide residues in grapes, and other fruits, which accelerates the development of agro-products.

## 5. Conclusions

Detection of pesticide residuals in agro-products is of significant importance for food safety. This study successfully identified pesticide residual levels of grapes using hyperspectral images at two different spectral ranges. The results showed that it was feasible to detect different residual levels treated by the mixtures of different pesticides which were in accordance with the real-world pesticide usage of grapes. Furthermore, to validate the performances of the HSI technology, three different varieties of grapes were studied, and all of them showed good performance. The comparison between conventional machine learning methods and deep learning illustrated the effectiveness of deep learning in pesticide residual level identification by HSI. More importantly, the wavelengths contributing more to the identification were identified by saliency maps of deep learning models, which was of great help to understand the spectral responses to the pesticides. This study illustrated that HSI can be used for pesticide residual levels identification. The non-destructive approach of HSI can be conducted in a contactless, rapid, and accurate manner, which improves the detection efficiency and reduces the costs and the use of chemical reagents. HSI can further be studied for on-line pesticide residual level identification. In future studies, a larger number of samples and more varieties of grapes should be studied to establish more robust models for real-world application. The optimization of deep learning models should be studied. Deep transfer learning can be used to improve the generalization ability of the established deep learning models. Furthermore, in addition to qualitative analysis, the quantification of pesticide residual content and the limit of detection (LOD) should be determined by HSI with deep learning methods. The mechanism of the active ingredients of pesticides on the spectral responses of grapes should also be studied.

## Figures and Tables

**Table 1:** Number of samples after cutting intact grapes.

Category	Cabernet	Red	Munage	Total
Level 0	73	92	89	254
Level 1	84	99	78	261
Level 2	60	107	104	271
Level 3	71	113	101	285
Total	288	411	372	1071

Level 1, Level 2, and Level 3 mean the pesticide mixtures with concentrations of 10%, 15%, and 50% prepared later, and Level 0 means distilled water.

**Table 2:** Information about the pesticides used in the experiment.

Category	Active Ingredients	Proportion	Efficacy
Jiatu	50% tebuconazole (C_16_H_22_ClN_3_O) 25% trifloxystrobin (C_20_H_19_F_3_N_2_O_4_)	4000	Brown spot
Huiyin	80% procymidone (C_13_H_11_Cl_2_NO_2_)	2400	Botrytis
Xishuangke	56% cymoxanil (C_7_H_10_N_4_O_3_) 14% cyazofamid (C_13_H_13_ClN_4_O_2_S)	6000	Downy mildew

**Table 3:** Information about the concentration of each pesticide in the mixture.

Concentration	Jiatu	Xishuangke	Huiyin
Level 0 ^a^ (0%)	0	0	0
Level 1 ^b^(15%)	0.0375	0.0250	0.0625
Level 2 ^c^ (30%)	0.0750	0.0500	0.0125
Level 3 ^d^ (50%)	0.1250	0.0834	0.2085
Standard solution(100%)	0.2500	0.1667	0.4167

^a^ means distilled water; ^b,c,d^ mean the pesticide mixtures with Level 1, 2, and 3, corresponding to concentrations of 10%, 15%, and 50%. The unit of concentrations is g/L.

**Table 4:** The classification of the accuracy of the logistic regression (LR), support vector machine (SVM), random forest (RF), convolution neural network (CNN), and residual neural network (ResNet).

Models	Categ	Parameter	Vis-NIR (%)			Parameter	NIR (%)		
			Train ^a^	Val ^b^	Test ^c^		Train	Val	Test
SVM	0	2.0, 0.1, poly	95.9	94.8	91.4	6.6, 1.0, linear	99.4	100.0	96.6
	1	1.2, 0.1, poly	98.4	96.3	92.7	1.0, 1.0, poly	100.0	100.0	96.3
	2	1.0, 1.0, poly	1.00	88.0	93.2	1.0, 1.0, poly	100.0	100.0	95.9
LR	0	1 × 10^5^, liblinear	100.0	89.7	93.1	100, lbfgs	99.4	93.1	98.3
	1	1 × 10^5^, liblinear	100.0	98.8	93.9	1 × 10^5^, liblinear	100.0	100.0	100.0
	2	1 × 10^4^, liblinear	100.0	92.0	95.9	100, newton-cg	100.0	98.7	97.3
RF	0	8, 450	100.0	77.6	79.3	6, 750	100.0	74.1	81.0
	1	7, 500	99.6	72.3	73.2	5, 550	98.8	86.7	87.8
	2	8, 200	100.0	66.7	75.7	4, 250	99.1	98.7	93.2
CNN	0	500, 32, 0.001	99.4	98.3	93.1	500, 32, 0.001	100.0	100.0	98.3
	1	500, 32, 0.001	97.6	97.6	92.7	500, 32, 0.001	100.0	100.0	98.8
	2	500, 32, 0.001	100.0	98.7	93.2	500, 32, 0.001	99.5	100.0	98.6
ResNet	0	1000, 32, 0.005	100.0	94.8	93.1	600, 32, 0.005	100.0	93.1	86.2
	1	1000, 32, 0.005	100.0	100.0	98.8	1000, 32, 0.005	100.0	100.0	97.6
	2	1000, 32, 0.005	100.0	97.3	94.6	600, 32, 0.005	97.7	100.0	97.3

^a,b,c^ represent training, validation, and test sets for the model; 0,1,2 represent Cabernet, Red grape and Munage, respectively, *Categ* mean Category of the grape. Parameters of the SVM, LR, RF, and CNN ResNet are shown. The parameters of the SVM, are (*C*, *gamma*, *kernel*); those of the LR are (*C*, *solver*); those of the RF are (*n_estimator*, *max_depth*); those of the CNN and ResNet are (epoch, batchsize, learning rate).

## Data Availability

The data presented in this study are available on request from the corresponding author.

## Supplementary Materials

The following supporting information can be downloaded at: https://www.mdpi.com/article/10.3390/foods11111609/s1, Figure S1: PCA score plot for the Cabernet, Red grape, and Munage spectral images photographed by using the Vis-NIR and NIR spectrometers.
