# Supplementary material for: Detection of Pesticide Residue Level in Grape Using Hyperspectral Imaging with Machine Learning
Source: Foods. 2022 May 30;11(11):1609. doi: 10.3390/foods11111609 (PMC9180647; doi:10.3390/foods11111609)
Supplement: Supplementary file 1 [file foods-11-01609-s001.zip › foods-1740871-supplementary.pdf]

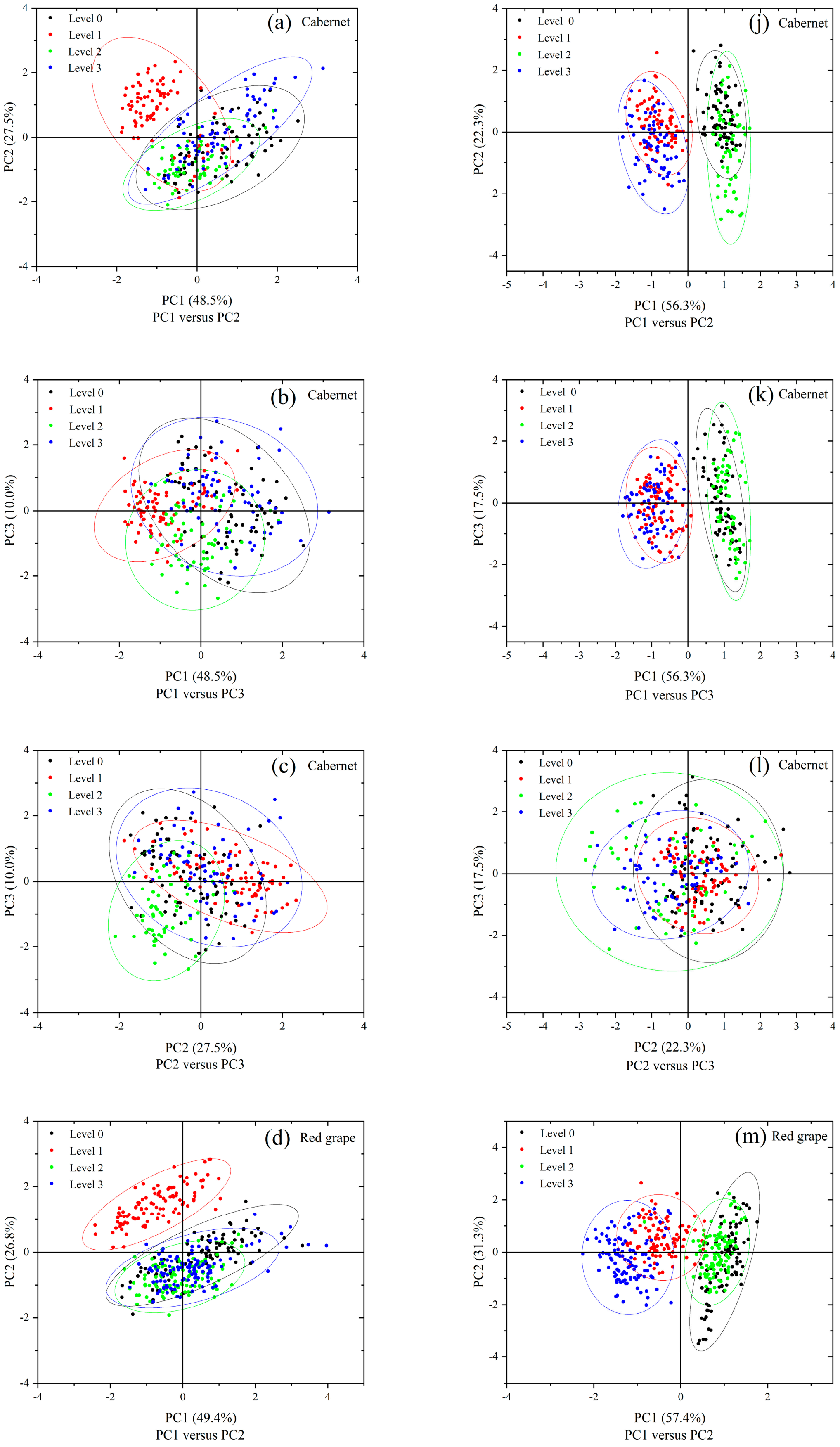

(a) Cabernet
Level 0
Level 1
Level 2
Level 3
PC2 (27.5%)
PC1 (48.5%)
PC1 versus PC2
(j) Cabernet
PC2 (22.3%)
PC1 (56.3%)
PC1 versus PC2
(b) Cabernet
PC3 (10.0%)
PC1 (48.5%)
PC1 versus PC3
(k) Cabernet
PC3 (17.5%)
PC1 (56.3%)
PC1 versus PC3
(c) Cabernet
PC3 (10.0%)
PC2 (27.5%)
PC2 versus PC3
(l) Cabernet
PC3 (17.5%)
PC2 (22.3%)
PC2 versus PC3
(d) Red grape
PC2 (26.8%)
PC1 (49.4%)
PC1 versus PC2
(m) Red grape
PC2 (31.3%)
PC1 (57.4%)
PC1 versus PC2

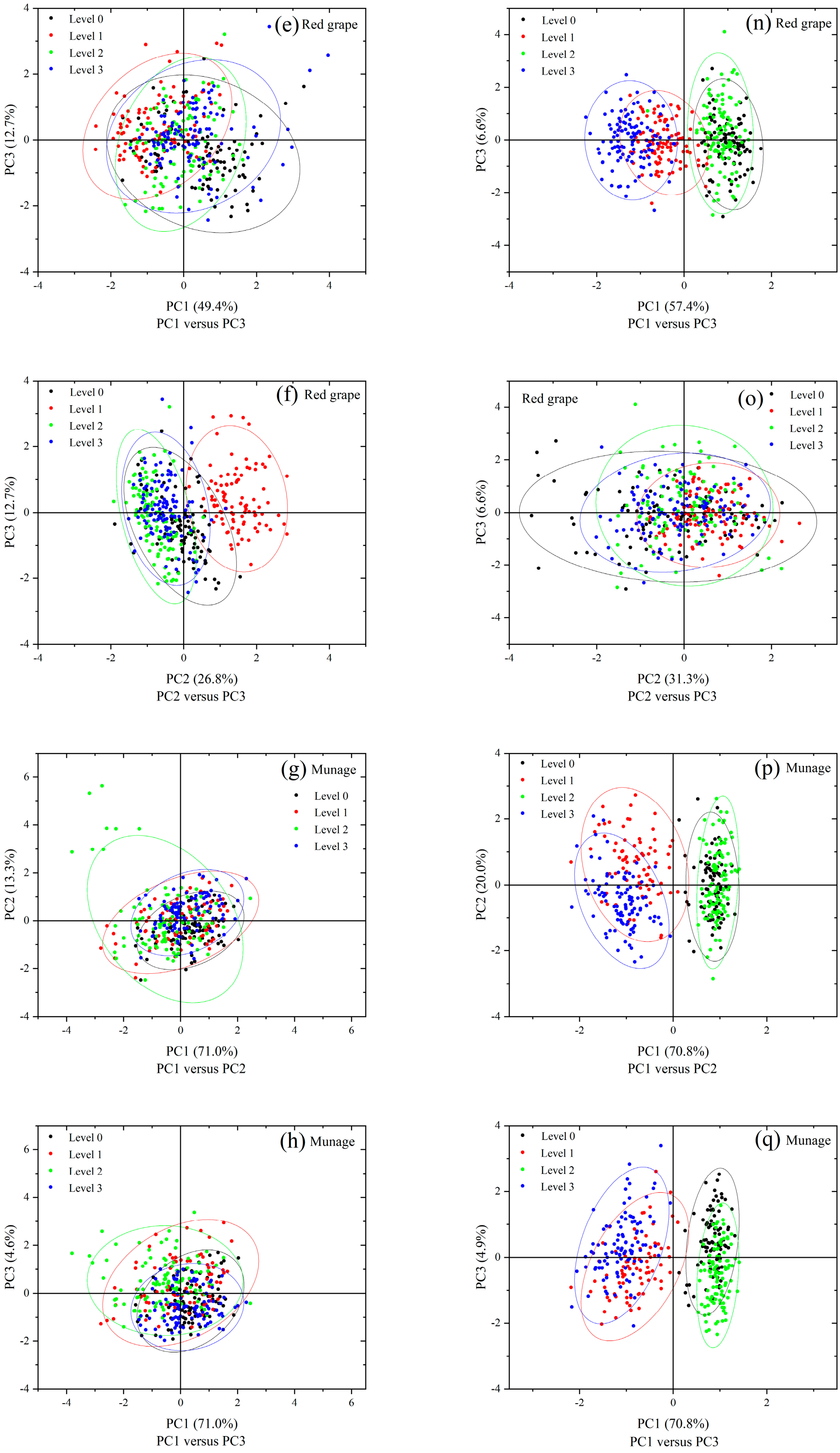

(e) Red grape
Level 0
Level 1
Level 2
Level 3
PC3 (12.7%)
PC1 (49.4%)
PC1 versus PC3
(n) Red grape
Level 0
Level 1
Level 2
Level 3
PC3 (6.6%)
PC1 (57.4%)
PC1 versus PC3
(f) Red grape
Level 0
Level 1
Level 2
Level 3
PC3 (12.7%)
PC2 (26.8%)
PC2 versus PC3
Red grape
(o)
Level 0
Level 1
Level 2
Level 3
PC3 (6.6%)
PC2 (31.3%)
PC2 versus PC3
(g) Munage
Level 0
Level 1
Level 2
Level 3
PC2 (13.3%)
PC1 (71.0%)
PC1 versus PC2
(p) Munage
Level 0
Level 1
Level 2
Level 3
PC2 (20.0%)
PC1 (70.8%)
PC1 versus PC2
(h) Munage
Level 0
Level 1
Level 2
Level 3
PC3 (4.6%)
PC1 (71.0%)
PC1 versus PC3
(q) Munage
Level 0
Level 1
Level 2
Level 3
PC3 (4.9%)
PC1 (70.8%)
PC1 versus PC3

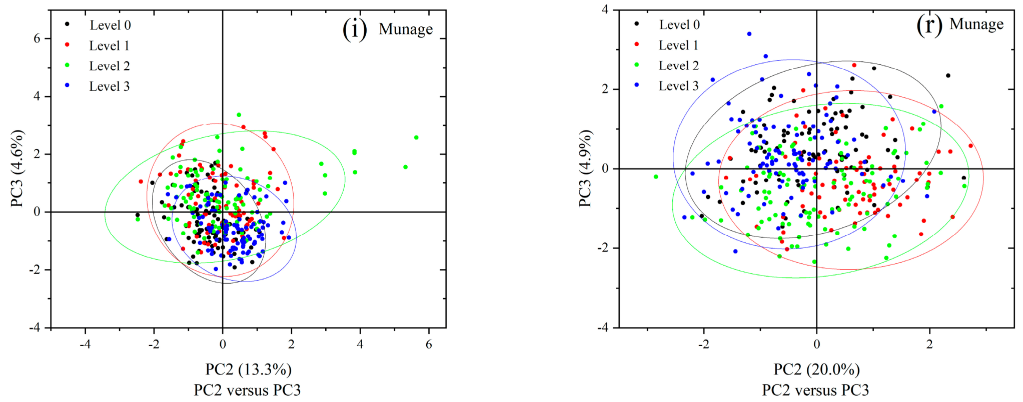

Figure S1. (**a–c**), (**d–f**) and (**g–i**) represent the PCA score plot for the Cabernet, Red grape and Munage spectral images photographed by using the Vis-NIR spectrometer. (**j–l**), (**m–o**) and (**p–r**) represent the PCA score plot for the Cabernet, Red grape and Munage spectral images photographed by using the NIR spectrometer.
